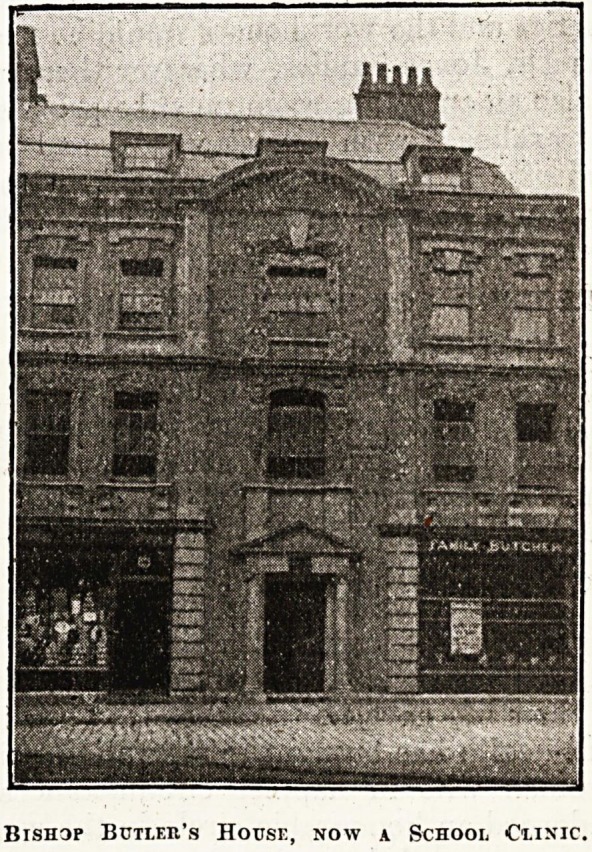# Hospital and Institutional News

**Published:** 1913-11-08

**Authors:** 


### HOSPITAL AND INSTITUTIONAL NEWS.

### THE PUBLIC AND THE MATRONSHIP AT YORK.

As regards the change in the matronship of York County Hospital, the *Yorkshire Herald* of Monday last, November 3, contains the following: " It is intimated that Miss E. Tute, the matron of the York County Hospital, has sent in her resignation on account of ill-health. It is a curious illustration of the manner in which the business of the hospital is conducted that the first announcement of this important matter is conveyed in a circular that has been issued on behalf of the house committee and the medical board in furtherance of a testimonial to Miss Tute. It appears that the resignation was intimated at a meeting of the joint bodies on Tuesday, October 28, and the opinion may be ventured that after what has happened recently in connection with the hospital a change of the nature involved by Miss Tute's resignation ought not to have been withheld from the public for a week, and then only intimated indirectly by a circular asking for subscriptions towards a testimonial." On such a relation as is here shown between a voluntary hospital and its subscribers no further comment than the local paper itself makes is necessary. How long, again we ask, will the public of York permit it to endure?

### NEW COMMISSIONERS UNDER THE MENTAL DEFICIENCY ACT.

The King, on the recommendation of the Home Secretary, has appointed Sir William Byrne, K.C.V.O., C.B., Assistant Under-Secretary of State for the Home Department, Dr. Rotherham, M.A., M.B., Medical Superintendent of the Darenth Industrial Colony, Dartford, and Miss Mary Dendy, Hon. Secretary of the Lancashire and Cheshire Society for the Permanent Care of the Feeble-Minded, to be Commissioners (paid) under the Mental Deficiency Act, 1913. Our readers will remember that both Dr. Rotherham and Miss Dendy were responsible for articles in our Special Mental Hospital Number, which was published on October 11 last. Both the new paid Commissioners, whose salaries under the Mental Deficiency Act are not to exceed £1,500 a year, are of course experts in their subject. Dr. Rotherham's knowledge and experience in dealing with "improvables" was made abundantly plain in the exhaustive interview he generously gave to our Commissioner last month, and, previous to his work there, Cambridge, St. Thomas's Hospital, and assistant medical officer's work at Horton and Cane Hill outline his professional career. Miss Dendy, the well-known protagonist of industrial colonies—more or less similar to the Darenth institution—has given public support to the Act by testifying from her experience against the cries of those who fear that individual liberty is not sufficiently safeguarded. Her article on "The Feeble-Minded as Producers" in our recent issue was remarkable for its record of the good accomplished by the colony treatment. Four unpaid Commissioners are to be appointed later.

### THE NEW BOARD OF CONTROL.

Under the Mental Deficiency Act the new Board of Control, which came into existence last Saturday, assumes a position of great importance. The existing Lunacy Commissioners are *ex-officio* members, and Sir William Byrne, K.C.V.O., C.B., has been appointed chairman. Since 1884 he has been connected with the Home Office, to which he was made Assistant Under-Secretary in 1908. In 1904 he was appointed a member of the Royal Commission on the Feeble-Minded. The Board will be composed of: Sir William Byrne, chairman, Dr. C. H. Bond, M.D., Dr. E. M. Cooke, M.B., Dr. S. Coupland, M.D., Miss Dendy, Mr. B. T. Hodgson, Mr. S. J. F. Macleod, K.C., Dr. F. Needham, M.D., Dr. A. Rotherham, M.B., Mr. L. L. Shadwell, Mr. A. H. Trevor.

### THE POOR-LAW ORDERS COMMITTEE.

Mr. John Burns, as President of the Local Government Board, has appointed Mr. A. V. Symonds and Mr. A. B. Lowry to act as additional members of the Departmental Committee on the Poor-Law Orders. Another change in the *personnel* of the Departmental Committee is seen in the appointment of Mr. E. F. C. Mosse to be one of its secretaries in the room of Mr. H. W. S. Francis.

### THE WORKHOUSE COMEDY AT NEWMARKET.

The comedy at Newmarket Workhouse has happily concluded, and the Rev. H. A. Douglas-Hamilton, the workhouse chaplain, is the popular victor of this conflict of circumstances and wills. At a meeting of the Newmarket Guardians on Tuesday, the Chairman reiterated his statement that the King's cheque, sent for the harvest tea (which had been discontinued unknown to the Chaplain at the time he applied to the King), had been not refused but unapplied. Mr. Douglas-Hamilton recalled a statement of the facts, such as, affecting him, we described last week, explained that he, as a new official, could hardly know that the treat had been discontinued, and justified himself quietly and well. The result was that the King's cheque will be received loyally; it will be spent in a treat, and everyone now should be pleased. The Guardians might have thus tactfully disposed of the matter before, and in their attempt to snub their Chaplain by repudiating his action, found that their last state was worse than his own. It is a victory for the Chaplain, but let us admit that the Guardians have made a graceful retreat.

### THE GRIEVANCES OF THE SCHOOL INSPECTOR

Medical inspection is a field which can only be satisfactorily cultivated after years of patient study, and that is why some share in the treatment of the discovered defects should decidedly fall to the lot of the medical inspector. Such work would not only vastly increase his interest in the inspection, but by affording an opportunity for increased salary and by relieving him of part of the routine medical inspection would enable the authority to retain his services over a long series of years. The importance of continuity of service in the case of inspectors is not fully recognised by the public, while, on the other hand, the question of treatment looms larger in the public eye. By allowing the medical inspector, therefore, to undertake treatment at the school clinic, and by giving him an assistant to help in the routine work, he can expect an increase of salary and a decrease of monotony, and could then with advantage to himself continue in the school service. It must be apparent that the inspector who is enjoying the experience gained from the treatment of defects is in a better position to advise parents as to the necessity for treatment or the continuation or repetition thereof than the inspector who takes no active share in the treatment. The monotony of school inspection has been greatly exaggerated. The examination of hundreds of children affords an almost unlimited experience in diagnosis, and cannot be described as an unduly restricted vocation for the competent inspector. But the tacking on to the work the opportunity of ameliorating and curing some of the defects which he has himself discovered cannot fail to counteract any latent tendency to monotony. This would also attract good men to the service who will be content to continue for many years perfecting themselves in this branch of the Public Health Service.

### NEW DIRECTOR-GENERAL OF THE ARMY MEDICAL SERVICE.

THE KING has appointed Surgeon-General A. T. Sloggett, C.B., C.M.G., K.H.S., to be Director-General of the Army Medical Service in place of Surgeon-General Sir Launcelotte Gubbins, K.C.B., M.V.O., M.B., K.H.S., when the latter vacates the appointment. For the past two years the new Director-General has been Director of the Medical Services in India, and has seen active service on the Frontier in 1884, while in 1896 he was senior medical officer of the British troops. He also served in the Nile Expedition, and was severely wounded in the battle of Khartoum. During the war in South Africa he had control of the Imperial Yeomanry Hospitals, and became principal medical officer of the General Hospital in the Deelfontein District. For his services he received the C.M.G., with medals and clasps. For the last ten years he has divided his time between England, as Principal Medical Officer of the Home District, and work in India of a similar kind.

### A PRIVATE COMMISSION ON THE BIRTH-RATE.

WE dealt only recently in a leading article with the true perspective of that much misunderstood question, the declining birth-rate, and can only now record the formation of a private commission of inquiry which the National Council of Public Morals has established. The composition of this privately formed body is somewhat curious; the clergy and bishops are well to the fore, but there is a medical element, including Sir A. Pearce Gould, Sir J. Crichton-Browne, Dr. Mary Scharlieb, and so on. Dr. T. H. C. Stevenson, the Superintendent of Statistics for the Registrar-General, and Dr. A. Newsholme, Medical Officer to the Local Government Board, have received Mr. Burns's consent to join the commission; but as they hold themselves free not to sign any public report we may take it that their presence is in the nature of a scientific corrective. For the question is a scientific rather than a moral one, and were it not that the pretentious title of a private unofficial body which calls itself a "National Council of Public Morals" excites little respect in scientific circles, it might be asked why Dr. Havelock Ellis, England's most distinguished publicist on this and similar questions —our one publicist, indeed, in this department who has gained the ear of Europe—is conspicuous by his absence. Fortunately, his views are known, and accessible in his "Task of Social Hygiene" and elsewhere.

### THE HAMPSTEAD HOSPITAL'S MOTOR AMBULANCE.

A REPORT has been issued on the working of the motor ambulance belonging to the Hampstead General and North-West London Hospital during the quarter ended September 30. The ambulance was presented, it will be remembered, by the Grand Duke Michael, and the London County Council decided to make a grant of £300 a year towards its maintenance on condition that it was available for street accidents. The number of direct calls by the police for the ambulance during the quarter was twenty-four, all of which were street accident cases except four. Twenty-two other cases were dealt with also, but the calls were not actually received by the police and were not made on the special telephone. The ambulance has been used in addition for thirty-seven surgical and thirty medical cases, a total for the three months of 113 cases. In every case the ambulance left the hospital within two or three minutes of the receipt of the call. The London County Council have suggested to the Commissioner of Police that the service might be further improved if the police on point duty were instructed to facilitate the passage of the ambulance, as at present it has to await its turn with ordinary commercial traffic.

### A CENTRAL MEAT STORE IN PROSPECT.

THE question of a central store for meat and other provisions has often been debated in relation to voluntary hospital finance, but it has always broken down through the difficulties of putting into practice such a scheme while the different institutions to be affected by it are run and administered on different lines. Such difficulties, however, do not apply in anything like the same degree to the institutions under the Metropolitan Asylums Board, and therefore more than casual interest attaches to the motion of Mr. G. S. Elliott that the question of establishing a central meat store for supplying the various institutions with meat and other selected perishable provisions be referred to a committee for consideration and report. This was agreed to, and whatever may be the result of the committee's deliberations the proposal has such a chance as it has rarely had in this country at least of being carried out in practice. There is little need to remind our readers that the institutions of the Metropolitan Asylums Board are highly centralised, and centralised moreover, if we may so put it, in an individual way. Each institution grows more and more specialised in its work, and the work of each is more and more closely made a contributory part of the whole. Thus, while the institutions, say, at Leavesden, at Carshalton, at Darenth, are specialised in the class of patients that they accept, the Darenth colony is practically the works department of the whole Board. With forty-two institutions to cater for, under one administration, a central meat store, so attractive in theory, might well be able to become a practical success.

### DOCTOR'S ENORMOUS ESTATE.

Dr. Francis Gray Smart, M.B., F.S.A., F.L.S., F.R.G.S., of Bredbury, Mount Ephraim, Tunbridge Wells, lord of the manor of Combe Hay, Somerset, who died on April 7, aged sixty-nine, left estate, "so far as at present can be ascertained," of the gross value of £446,819, of which the net personalty has been sworn at £445,254. The duties are expected to be over £70,000. Dr. Smart's wife, who died a week before he did, left gross estate of £750,443. Dr. Smart's bequests include £10,000 to the London Homœopathic Hospital on trust for investment, the income to be devoted to current expenses, and £10,000 to the Homœpathic Hospital of Tunbridge Wells. Rich medical men are still so rare, and medical thousandaires, to use a perfectly correct expression, so rare also that the present instance is especially noteworthy.

### ANOTHER HOSPITAL FOR SALE.

The British Lying-in Hospital, Endell Street, St. Giles, founded in 1749, we are informed, will be sold by public auction at the Mart, Tokenhouse Yard, E.C., on the 20th inst., at 2 o'clock. The building is freehold property, consisting of four floors and basement, prominently placed at the corner of Short's Gardens. The Nurse's Home in Betterton Street, which will also be sold, communicates with the hospital, and is a fire-proof building of five floors and basement. The entirety has a total frontage of 182 feet to three thoroughfares, and occupies an area of 6,600 square feet. It is said to be pre-eminently adapted for a public institution or club.

### AN ALMONER ON OPEN-AIR SCHOOLS.

We have been favoured with a copy of the address which Miss Wilson, almoner to Leeds General Infirmary, recently delivered to the Yorkshire Branch of the Association of Teachers of Domestic Subjects on "Social Work from the Point of View of the Hospital." Having a lay audience, Miss Wilson naturally dealt rather with the broad and simple root problems of the almoner's work, and her careful picture of the out-patient department and of its failures through, for this is the main cause, the poverty or precarious employment of the families, is only too well known to almoners and the hospital world generally. The local authorities, however, were criticised by the speaker for general lack of co-operation, and chiefly for procrastination in providing an open-air school. On the need for this Miss Wilson strongly insisted. Bradford, of course, possesses one, but in arguing on their behalf it is most important to consider carefully whether a day open-air school or a residential open-air school is to be provided. When an institution of the former kind was opened in Paddington, about a couple of years ago, we drew attention to the fact that the advantage gained by an open-air day school is largely undone when the children return to their own homes for the night. The lesson, in fact, is that those in charge of open-air day schools inevitably regret this undone good, and end by clamouring for residential or boarding schools. That is probably what led Mr. James Graham, quoted at the time by the Yorkshire papers, to criticise the day school scheme.

### HISTORIC HOUSE AS SCHOOL CLINIC.

The historic house of Bishop Butler, situated in Kingsmead Square, Bath, and shown in the accompanying photograph, has recently been opened by

Bishop Butler's House, now a School Clinic.

the local authorities as a school clinic. The clinic is in the upper part of the building, and it appears that shops have taken possession of some part of the ground floor.

### NEW LADY ALMONER AT NORWICH.

The recent appointment of Miss C. M. Smyth to be lady almoner at the Norfolk and Norwich Hospital is interesting as marking the close of a successful period of work in this revived office on the part of her predecessor, who resigned last month. Miss Smyth has been acting as assistant to the late almoner, Miss E. R. Waley, who, for nearly seven years, for she was appointed in January 1907, has controlled the work of the department from the time of its inauguration at that date.

### OPERATIONS IN SMALL POOR-LAW INFIRMARIES.

Dr. J. Owen Jones, the workhouse medical officer to the Holywell Board of Guardians, has made some interesting criticisms of their new infirmary, which has been built, at a cost of £7,000, by Mr. Davies, of Chester. Apart from the site, to which he objects, Dr. Jones has stigmatised the lack of an operating-room and a mortuary as grave omissions. In reply the architect, Mr. Davies, states that the Local Government Board did not wish to encourage the performance of major operations in the small provincial infirmaries, and also that it was not always advisable to have a mortuary close to the institution, though one could be built if the Guardians wished. The fact is, of course, that so long as workhouses are not completely separated from infirmaries—the latter should be independent institutions with a separate staff—such difficulties will always arise. There should be no such thing as workhouse sick wards. The sick should be in a separate infirmary, and every infirmary must, of course, have its theatre and its mortuary. In order, however, to make this separation complete a rearrangement of the small infirmaries and the workhouses would be necessary, but, as Dr. Jones implies, wherever there are sick, there also an operating-room must be provided. The small workhouse with its sick wards, and part-time medical officer, is the great drawback in the Poor-Law medical service, and must be done away with before long.

### READING FOR WORKHOUSE INMATES.

The gift of £5 from Lord Rosebery to the Epsom Guardians for the purchase of literature for the use of the inmates of the workhouse is a matter of more importance than the casual observer might think. It brings forward the conviction, on the part of a notable man of letters, that "man"—even if he be the poorest of men—"does not live by bread alone." The inmates of our workhouses are not all degraded—not even all dull. Some, indeed, have fallen into dire poverty through qualities for which their life gave no true scope; while in other cases ill-health has brought to starvation point men and women who in happier circumstances might have adorned an honourable circle in the world. If instances are needed one has but to quote the fact that Francis Thompson stood in the gutter, selling the oddments of the hawker, and that the author of the words of that popular song, "The Rosary," is even now the inmate of a charitable, though not a Poor-Law, hospital. A very little worse turn of fortune might have sent either of these two into the workhouse. We do not pretend that every workhouse inmate is of this calibre, nor even many of them. But in a workhouse one finds, as those who visit it will admit, men and women of every grade of coarseness and of refinement. All, under the necessary administration of the law, must be treated equally, and it is not of such matters as food or of necessary rules and regulations that the better class complain. But they are lonely among their surroundings, and they are deprived of such consolations as they might find in books. Where old magazines are given for the use of the inmates of a workhouse they are deeply appreciated. We remember seeing cherished volumes of *Home Chat* (really odd numbers bound in the workhouse by some of the inmates) read most lovingly by old women who never hoped to see, far less wear, any of the garments illustrated in the journal; while stray numbers of the *Strand Magazine* were nearly worn out with much handling. Old age always needs special consideration, and when old age is combined with the direst poverty such alleviation of its conditions as is given by the provision of something to read is very precious. We are grateful to Lord Rosebery for bringing this before the notice of the public.

### NEW DEVELOPMENTS AT THE ROYAL FREE HOSPITAL.

The most interesting statement at the quarterly meeting of the committee of management of the Royal Free Hospital, over which Sir E. Durning Lawrence, Bart., presided, was that it is hoped that the new out-patient department will be opened during the first half of 1914. The accommodation to be provided therein is urgently needed to cope with the increasing demands upon the hospital's accommodation. In fact, during the past quarter a considerable increase has been noted in the treatment of gynæcological and throat, nose, and ear patients. It is also interesting to note that negotiations have been proceeding for some months past for securing recognition of the hospital as a centre for treatment of tuberculosis under Section 16 of the National Insurance Act. The importance attached to the retention of the treatment of tuberculosis cases through the medium of hospitals is a matter, it is felt, that should be fully impressed upon the authorities, who should realise that what is known as dispensary treatment is often in practice little more than out-patient treatment, and it is only by securing the co-operation of the general hospitals and the special hospitals for consumption that the spread of disease can be properly checked.

### INSTITUTIONAL TREATMENT OF THE UNINSURED.

A fortnight ago in The Hospital reference was made to the progress of the London County Council's scheme for the treatment of tuberculosis in the Metropolis, as far as the provision of dispensary treatment was concerned. The Public Health Committee of the Council have now started to tackle the question of the arrangements for the provision of residential accommodation for uninsured persons. From the outset the Local Government Board has made it clear that it is of opinion that the County Council should provide, or arrange for the provision of, all residential accommodation required for the treatment of tuberculous persons in London. Now that Parliament has by legislation removed the barrier which prevented the Metropolitan Asylums Board from taking a direct part in the work, the

Public Health Committee are urging that any comprehensive scheme for dealing with the treatment of tuberculosis in London should provide that the County Council shall make arrangements with the Asylums Board and with hospitals and sanatoria for the provision of the residential accommodation required, and the Committee propose to consider and report whether adequate accommodation can be provided in this manner without the Council being called on itself to own and manage institutions. The Finance Committee of the Council state that there is at present no reliable information as to the number of beds which would be required, and at the present stage no estimate can be given of the expenditure which the scheme will ultimately involve. But at present, of course, the Council is considering general principles and not detailed schemes. At a later stage the whole question will again have to be very carefully considered, when the Council has some idea of the amount which is likely to fall upon the London rates. That will depend very largely upon to how great an extent the Asylums Board and the voluntary hospitals will be prepared to assist the Council in their work.

### DEATH OF DR. FRANKLIN PARSONS.

We regret to record the death of one of our contributors, Dr. Henry Franklin Parsons, formerly of the Medical Department of the Local Government Board. Dr. Parsons, whose striking series of articles on Isolation Hospitals and their Work is still to be concluded, was born in Somerset, and studied at St. Mary's Hospital, where he gained many distinctions, including the University gold medal in physiology, anatomy, and comparative anatomy. In 1876 he also gained the gold medal at the examination in public health, and later became examiner to Cambridge University for its D.P.H. He soon abandoned private practice for the post of Medical Officer of Health for Goole and Selby, and after five years became a medical inspector of the Local Government Board, where he remained till 1911, the year of his retirement. This long period of work was largely filled by service on numerous Departmental Committees, including those on regulations for cremation; water; gas; geological survey; and the medical inspection and feeding of school children. He was president at one time of the Geological Society, and a frequent writer on epidemiology, cemeteries, and other allied subjects. That he was also a remarkably lucid and well-informed writer our readers can testify. He was sixty-seven years of age.

### HOSPITAL TREATMENT OF INSURED IN LONDON

Signs are not wanting that the relation of the voluntary hospitals to National Insurance patients may shortly undergo revision, and fulfil to the mutually greater advantage of themselves and of the insured patients the due position as a centre for consultation and special treatments. The London Insurance Committee, on the motion of Dr. H. H. Mills, recently referred to the Medical Benefit Sub-Committee "to inquire and report as to the facilities for obtaining clinical investigations and treatment for insured persons at the Metropolitan Hospitals." An instance of the change adumbrated may be found in the agreement of the Public Health Committee of Kensington to supply its district panel doctors at the expense of the rates with the necessary outfits for the collection of specimens in all cases of suspected tuberculosis, venereal disease, typhoid, and diphtheria, the bacteriological examinations necessary to be made at the Lister Institute. The immediate result may be a Conference of the London Hospitals with the London Insurance Committee, and the holding of such a Conference would be in the highest degree interesting.

### THE INSURED AND DR. DIMOCK.

The death of Dr. Dimock after proceedings of criminal libel had been instituted, which we mentioned last week, was followed by a popular demonstration of sympathy organised, it is curious to note, by a committee of insured workers. Resolutions were passed thanking the medical men who supported him, and expressing satisfaction that the Commissioners had decided to keep the panel closed in Wisbech and welcoming the appointment of a new doctor. One speaker affirmed that the deceased doctor had been persecuted since his arrival. Meanwhile the windows of non-panel doctors' houses were broken. Some two hundred representatives of insured workers went to his funeral, and Dr. Coffey, special panel doctor at Chatteris, Dr. Beckett, Dr. Price, of Upwell, and Dr. Howe were present, and on the representatives' return to Wisbech noisy scenes occurred. The crowd hooted outside Dr. Meacock's residence, broke a window in Dr. Gunson's, and were only prevented by the police from returning to annoy Dr. Meacock further. As the complainant in the libel action he was the chief object of their attack, and forty panes of glass were broken, while his front door received serious damage. Indeed, matters at one time became so serious that the Mayor, who had been specially summoned, read the Riot Act, and on the people refusing to disperse the police drew their batons and charged the crowd, several of whom, in the old phrase, must have had cracked sconces. Let no rash politician, we may add in conclusion, quote this demonstration as a proof that panel men and medical blacklegs are the finest flower of their profession, for though Dr. Dimock has now passed where no further criticism would wish to follow, it must be stated that in his case such a claim could not have been made out.

### SCARLET FEVER ACCOMMODATION.

Attention was called at a meeting of the Southwark Board of Guardians on Thursday last week to over twenty cases of scarlet fever, many of which had remained in their homes for twenty-four hours. Mr. J. Osborn, the vice-chairman, had attempted to get some of the worst cases removed, especially those where families of six and eight people were living in two rooms. He was told by the officials of the Metropolitan Asylums Board that the hospitals were full up. Mr. Osborn rightly contended that they expected the various hospitals for contagious fevers to be in readiness for a periodical outbreak, and they lived in a fool's paradise if at such a time they were told that institutions were full, and that nothing could be done. He understood that one reason why there were no beds was because the Metropolitan Asylums Board had allocated them for tuberculosis patients under the National Insurance Act. Mr. T. Cornell, a member of the Metropolitan Asylums Board, replied that it was not a question of accommodation at all, but a question of the dearth of nurses. The Board had beds for 6,000 to 7,000 scarlet-fever patients, and were doing all they could to relieve the wants of London generally. They had 180 spare beds at Joyce Green Hospital, Dartford, but they had no nurses. They had received offers of the help of "sisters," but what they required was workers. He thought at such a crisis as this there might be ladies willing to help, as they had done on previous occasions. The term workers is ambiguous, for presumably no one without training in a particular capacity would be employed at all. Perhaps Mr. Cornell will explain.

### OUT-PATIENT CONDITIONS AT LEEDS INFIRMARY.

As a result of watching "for a couple of hours the children just out from the adenoid operations in a screened-off corner of the out-patients' waiting-room" of the General Infirmary, Leeds, Mrs. E. M. Marvin has urged in the Yorkshire papers the need for the presence of women on the infirmary board. In reply to this Mr. Charles Lupton, the honorary treasurer, has publicly stated that the large number of operations performed create a difficulty, but that there is no reason why children should leave the institution in the condition—vomiting blood—that Mrs. Marvin described. Mr. Lupton adds that the present difficulty, on which public light has been thrown by this lady, is one of the chief reasons why the board is asking money to extend the out-patient department. Various schemes are before it for rearranging the work, and, with the necessary money, whatever ground of complaint there may be will be done away with. The interesting question remains, however, whether Mrs. Marvin, whose action has admittedly brought the hospital's difficulties into the limelight, would have been as free with her adjectives if she had been a member of the board. What is our readers' opinion?

### NEW SUPERINTENDENT AT ABERDEEN.

Dr. James Chalmers, of Aberchirder, Aberdeen, has been appointed superintendent of the City Hospital, Aberdeen. He began his career by being apprenticed to a pharmacist, and then passed to Aberdeen University, where he gained several distinctions, and graduated D.Ph. last year. For the past five months Dr. Chalmers has acted as principal assistant under Dr. Matthew Hay, at the City Hospital, and, therefore, his new appointment is a promotion in his connection both with Aberdeen and with this particular institution.

### THE INSURANCE ACT AND DISPENSING.

The contracts between the panel chemists and the local Insurance Committees will shortly be renewed, and chemists are now considering the question of revising the tariff of prices for drugs and appliances upon which these contracts will be based. A proposed new tariff has been drawn up by the Pharmaceutical Standing Committee on Insurance and submitted to the local Pharmaceutical Associations concerned, and it is this draft which is now being discussed. Since the existing tariff was first adopted the prices of many of the drugs included therein have altered considerably, and it has been necessary to change the tariff rates accordingly. The principle upon which the new tariff is based is the same as that adopted in the case of the first tariff, a charge being made for each ingredient of a compounded medicine according to its quantity and value and an additional fee for the time and skill required for dispensing. A number of alterations has, however, been introduced and prices for various quantities have been carried out in greater detail. Many additions have been made to the list of drugs and preparations, but it is, of course, open to Insurance practitioners to prescribe any drugs not included in the list, although it is left to the local Insurance Committees to decide whether or not proprietary preparations may be charged to the Drug Fund. In many districts there is a probability that the funds at the disposal of the Committee will not suffice to pay the bills of the chemists in full, and in these districts the Committees are prohibiting the prescribing of proprietary drugs on account of their greater cost.

### THIS WEEK'S DRUG MARKET.

An improvement in business cannot be long delayed, but at present the demand for drugs cannot be called brisk. Even the market for cod-liver oil is almost lifeless, notwithstanding that the chief consuming season is so near at hand; in consequence of the continued absence of demand prices are tending lower, but it is not improbable that they will improve again when business becomes active. A fair business is being done in opium at unchanged rates; morphine has a slightly lower tendency, and codeine is unchanged; any further reduction in the value of opium might, however, be followed by a reduction in the price of the latter alkaloid. The value of citric acid is well maintained, and may advance still further. Cascarilla bark is very scarce, and dearer. The price of oil of cloves is lower. Manna is cheaper. Eucalyptus oil is unchanged in price, but it is not improbable that an advance will take place when the demand becomes more active. Essence of lemon continues to decline in value. Castor oil tends downwards in price. The value of hydrastis canadensis tends upwards. Spermaceti has a downward price tendency. At the recent public sale of drugs in Mincing Lane there was some improvement in the demand. Cape aloes sold at lower rates, but prices are still high. Cascarilla bark fetched very high prices Cardamons were again rather dearer.

## Figures and Tables

**Figure f1:**